# Changing the Tone of Clinical Study Design in the Cannabis Industry

**DOI:** 10.1515/tnsci-2020-0002

**Published:** 2020-02-13

**Authors:** Joseph M Antony, Alison C. McDonald, Farshid Noorbakhsh, Najla Guthrie, Mal Evans

**Affiliations:** 1 KGK Science London, London Canada; 2 Department of Immunology, School of Medicine, Tehran University of Medical Sciences, Tehran, Iran

**Keywords:** cannabinoid, brain activity, circadian rhythms, clinical trials

## Abstract

Cannabis (also known as marijuana) is the most frequently used psychoactive substance globally. Cannabis exerts therapeutic functions for many indications and has vast potential as a health and wellness product. Advances in our understanding of the composition and pharmacological properties of cannabis have revealed interactions between cannabis, an individuals’ circadian rhythms and their endocannabinoid signaling. Exogenously administered cannabinoids can bidirectionally entrain central and peripheral clocks that comprise circadian rhythms, and malfunctions in the endocannabinoid system are reported to impact neurological processes. Therefore, it is necessary to account for the circadian rhythm when designing clinical trials examining the pharmacological properties of cannabis-based products for health and wellness to limit its potential confounding impact on results. Consideration of the entrainment capabilities of the endocannabinoid system is warranted when designing clinical trials.

## 1 Introduction

Clinical trials that do not factor an individual’s circadian rhythm when investigating the efficacy of cannabinoids on various health outcomes may be at a disadvantage, resulting in irreconcilable results. This paper focuses on the need for synchronizing circadian rhythms in the study population when designing clinical studies to optimize intervention outcomes and avoid confounding variables arising from faulty trial design. By incorporating the circadian rhythm-controlled endocannabinoid system (ECS) into clinical trials in the cannabis space, we aim to inform clinical trial designs that are best able to demonstrate how cannabis health products can impact health and wellness.

### 1.1 The endocannabinoid system

The ECS is a complex cell signaling system that is involved in the regulation of several crucial functions throughout the body. The ECS is composed of endocannabinoids, which are endogenous lipid messengers, two distinct G-protein-coupled receptors, i.e. type 1 and type 2 cannabinoid (CB1 and CB2) receptors, and enzymes responsible for the synthesis and inactivation of the endocannabinoids. The best-studied endocannabinoids are *N*-arachidonylethanolamine (anandamide; AEA) and 2-arachidonoylglycerol (2-AG), an abundant endogenous agonist of the CB1 receptor. Both are synthesized on demand from the membrane lipid precursors, *N*-acylphosphatidylethanolamines and diacylglycerides, respectively. Furthermore, there are endogenous bioactive lipids called *N*-acylethanolamines (NAEs), such as *N*-linoleoylethanolamine (LEA), *N*-palmitoylethanolamine (PEA), *N*-oleoylethanolamine (OEA) and *N*-stearoylethanolamine (SEA), which are also produced through the same biosynthetic pathway as AEA. Cannabinoid receptor activity is modulated by NAEs indirectly by interfering with endocannabinoid metabolism [1].

### 1.2 Cannabinoid receptors in the central nervous system

A majority of the CB1 cannabinoid receptors are widely expressed in the brain, particularly in the cerebral cortex, septum, amygdala, hypothalamus and parts of the brain stem and the dorsal horn of the spinal cord. These receptors are present in neurons and glia, where they mediate synaptic transmission. In the brain, CB1 receptors exert several functions that influence learning, memory, cognition, motor control, anxiety and depression, appetite and food intake, reward and addiction, neuroprotection, neural development and sleep [2]. Endocannabinoid signaling in the amygdala is involved in the regulation of stress, anxiety, and fear. For example, overexpression of the catabolic enzyme, fatty acid amide hydrolase, in mice rapidly hydrolyzed AEA resulting in reduction of stress, anxiety and conditioned fear expression [3]. Aberrations of the endocannabinoid system are increasingly recognized as etiological factors in anxiety and mood disorders due to the presence of CB1 receptors in key regions of the corticolimbic brain networks, such as the prefrontal cortex and amygdala, which functionally interact with subcortical dopamine pathways [4]. The ECS in the brain reinforces food consumption by interacting with the mesolimbic pathways involved in reward mechanisms, where this pathway is activated in the hypothalamus [5]. Further, the neuroprotective and anti-inflammatory roles of the endocannabinoid system predominantly mediated by inhibition of pro-inflammatory cytokines have been documented in various *in vitro* and *in vivo* models of neurological disorders [6].

### 1.3 The circadian rhythm

The term circadian was introduced in the 1950s to recognize self-sustained rhythms under constant conditions. Diurnal rhythms in physiological systems respond to environmental variables such as the light-dark cycle [7]. Circadian rhythms are 24-hour rhythms that are characterized by the behavioural and physiological changes from day to night, which are controlled by the pacemaker and its regulator, and coordinates internal time with the external world [8]. The principal region of the brain that coordinates the 24-hour biological rhythmicity is the suprachiasmatic nucleus (SCN) located in the hypothalamus [9]. The endogenous circadian rhythm consists of the master/server clock seated in the SCN and the subordinate/client clock which is embedded in nearly every cell in the form of interlocking transcriptional-translational negative-feedback loops made up of *Clock* genes that exert their biological effects via *Clock*-controlled genes [10]. At the genetic level, heterodimerization of brain and muscle Arnt-like 1 (*BMAL1*) with Clock or neuronal PAS domain protein 2 (NPAS2) activates the translation of the *PER* and *CRY* genes, whose mRNA accumulates in the cellular nucleus in the morning, but are heterodimerized in the afternoon in the cytoplasm, followed by phosphorylation by casein kinase1 that inhibits BMAL1-Clock heterodimer in the evening [9].

Circadian disruption exacerbates pathological events due to the role it plays in the fluctuation of the sleep-wake cycle, motor disability, and the autonomic nervous system. There are limited pharmaco-therapeutic options available for disrupted circadian rhythms. Resetting the circadian clock can be achieved by chronotherapy [11], exogenous melatonin [12] that is widely available as a nutritional supplement, and timed bright light exposure, considered as alternate strategies.

### 1.4 The endocannabinoid system is modulated by the circadian rhythm

Endogenous cannabinoid signaling is a mechanism regulating intercellular communication in the body. Endocannabinoid ligands link the output of the central circadian pacemaker in the SCN with physiological processes, such as appetite, feeding, peripheral metabolism, anxiety, and depression. Cannabinoid receptor signaling can be pharmacologically manipulated to affect sleep/wake cycles, temperature regulation, food consumption and fat storage, central nervous system regulation of autonomic and endocrine functions, reward-driven behavior, gastrointestinal function, mood, and sensory perception. Cannabinoid receptor agonists with diverse intrinsic activities and affinities for cannabinoid receptors include those that show affinity for CB1 and CB2 receptors [13]. Molecules that enhance cannabinoid action by inhibiting endocannabinoid uptake and blocking catabolic processes though these compounds often lack selectivity. Among antagonists of the CB1 receptor, rimonabant has been shown to improve lipid and glucose metabolism [14].

The activity of the ECS is also modulated by diurnal rhythmicity. It has been found that the expression pattern of CB1 and CB2 receptors are influenced by the light/dark cycle and therefore appears to be under the control of a diurnal rhythm, at least in normoglycemic Wistar rats [15]. Further, the ECS can potentially modulate circadian rhythms in rodents, demonstrated by the modulation of light-induced circadian rhythms of the hamster by intraperitoneal injection of the CB1 receptor agonist, CP55940 [16]. Evidence from healthy humans show that plasma concentration of the endocannabinoid, AEA, shows a circadian rhythm with concentrations being three times higher on waking than just before sleep and endocannabinoid signaling was found to be necessary and sufficient for the control of sleep stability, but not necessary for sleep homeostasis [7]. Sleep restriction amplifies the rhythm of plasma levels of 2-AG resulting in excessive food intake contributing to obesity [17] demonstrating that endocannabinoid activity is profoundly modulated by circadian rhythmicity [18]. Among healthy sleep-deprived men who underwent an exercise paradigm, there was an influence on appetite and mood corresponding to 80% higher pre-exercise plasma concentrations of 2-AG 90 minutes after awakening when compared to men who had normal sleep [19]. Importantly, these studies show that 2-AG concentration and timing of acrophase were influenced not only by the duration of sleep, but also time at sleep. This is an important aspect to consider when designing a clinical study, such that participants included have similar sleep schedules, and shift workers are excluded from the study. The evidence suggests interesting timed interactions within a biological system. Endocannabinoid levels fluctuate over the circadian cycle and experiments conducted over short time windows can result in conflicting results [20], as noted with CB1 antagonist-treated rodent model of sleep [21] or fragmented sleep observed in CB1-null mutant mice [22]. As a neuromodulator, the crosstalk between endocannabinoid and other neurotransmitter systems, via either local neural circuits, receptor heteromerization, or downstream signaling, has been established.

### 1.5 Interactions between the ECS and exogenous cannabinoids

The non-psychoactive constituent of *Cannabis sativa* L., cannabidiol (CBD), may enhance endocannabinoid signaling. Exogenous activation of the endocannabinoid receptors alters the level of endocannabinoids, an observation noted in healthy individuals who received a large intravenous dose of delta-9-tetrahydrocannabinol (THC), the primary psychoactive cannabinoid constituent of cannabis [23] and an endocannabinoid mimetic. Endocannabinoid plasma concentrations showed a biphasic response after THC injection with an initial spike followed by a significant decline and gradual return to baseline levels. Levels of endocannabinoids at 8 am and 3 pm were nearly identical with no significant differences in AEA and 2-AG between the two time points. Endocannabinoids reached maximum concentrations 30 minutes after THC administration and returned to baseline values at 300 min and 48 hours, while THC and THC- OH were measurable at 24 hours and remained detectable until 48 hours after THC dosing [24]. The augmented levels of endocannabinoids may be mediated by enhanced catecholinergic and glucocorticoid signaling, a direct pharmacologic effect of THC on endocannabinoid synthesis, or degradation. The significant decline in plasma endocannabinoids 30 minutes after THC administration, attaining baseline levels 48 hours after the study, may be due to decline in THC levels or that of its metabolites, which cause direct adrenal stimulation or sedation induced by the drug. Thus, repeated THC treatment may induce a biphasic endocannabinoid response characterized by an early symphatico-adrenergic and glucocorticoid activation during high plasma levels of THC followed by a relaxation response associated with lower endocannabinoid plasma concentrations [25, 26].

Thus, a circadian pattern in endocannabinoid signaling with a maximum in the morning and minimum in the evening was shown [18], whereas another study of healthy volunteers administered THC intravenously did not exhibit a circadian rhythm [24]. This may be attributed to the study design, since the former study included healthy volunteers who self-reported habitual sleep duration of 7.5 to 8.5 hours [18], whereas this was not accounted for in the latter study [24]. The diurnal nature of endocannabinoids and its impact on medicinal and nutritional interventions may have been overlooked within this paradigm, leading to challenges in making efficacy conclusions. Thus, sleep deprivation, which can increase the level of endocannabinoids and amplify hedonic food-seeking behavior, can be reversed using CBD acting preferentially on the CB2 receptors to achieve circadian biorhythm.

### 1.6 Effect of exogenous cannabinoids on the circadian rhythm

Exogenous cannabinoid consumption can distort perception of time due to modulation of the brain’s circadian clock, SCN, which expresses CB1 receptors. Entrainment is an essential adjustment of the circadian phase to the environment [7] and cannabinoids attenuate the ability of the circadian clock to entrain to light zeitgebers or cues. This effect is mediated at the level of altered gamma amino butyric acid (GABA)-ergic communication within the SCN. Activation of the CB1 receptor reduces the release of GABA from pre-synaptic axon terminals in the SCN thereby increasing neuronal SCN activity. Administration of exogenous AEA consistently increased rapid eye movement (REM) sleep and non-REM sleep, and as well, the CB1 antagonist/inverse agonist, rimonabant, reported sleep disturbances. Thus alterations in SCN timing is one mechanism by which cannabinoids influence time perception, in addition to effects in the cortex, hippocampus, or striatum [27].

CBD has an effect on circadian genes in the microglia, an immune cell in the brain. Microglial cells produce endocannabinoids at higher levels than neurons, *in vitro*, supporting the important role of activated microglia in neuroinflammation associated with cannabis. Chronic administration of THC leads to desensitization and downregulation of CB1 receptors and therefore to a higher tolerance regarding peripheral and central effects of the drug, which have been attributed to the dysregulated phosphorylation of the CB1 receptors. Chronic cannabis use could implicate an abnormal glia-neuron communication, as observed in a significant dysregulation of circadian genes, with upregulation in *Arntl* and downregulation of *Clock* genes. Circadian genes in microglia were dysregulated by CBD regardless of LPS stimulation due to the upregulation of *Arntl/Bma1*, which maintained microglia in a state of early awakening, whereas negative regulation of *Per1* blocks the natural cycle of the cell, maintaining the cell in perpetual morning state [28].

Pre-clinical studies have shown that cannabinoid treatment with a CB1 agonist can inhibit light-induced phase shifts by 90%, warranting the need for evaluation of adverse effects of cannabinoids on circadian rhythms in humans [16]. While there is no link between *Clock* genes and cannabis addiction, the expression of dopaminergic D2 receptor gene (*DRD2*) is *Clock*-controlled and may be involved in cannabis addiction by co-expression with the CB1 receptor gene [9]. Interestingly, circadian rhythm genes in microglia were dysregulated by CBD, but not by THC, consistent with its use to treat insomnia [28]. However, high (15 mg) dose of THC acts as a sedative in healthy volunteers while a similar dose of CBD has alerting properties, and counteracts the sedative properties of THC [29]. The outcomes observed with administration of CBD and THC may differ with the population studied (insomnia versus healthy) and could be attributed to the effect of endocannabinoids, justifying the need to examine their levels in clinical studies.

### 1.7 Clinical Trial Design: Controlling for the confounding effect of circadian rhythm on outcomes

Aligning interventions to circadian rhythms to enhance outcomes is common practise in the pharmaceutical industry. Adjustment to circadian rhythm is known to correspond to better treatment in laboratory animals subjected to leukemia [30]. This observation gave rise to the field of chronotherapy and subsequently, animal studies showed that circadian cycles were important in diseases in general. Among patients undergoing heart surgery, those who undergo surgery in the afternoon are less prone to post-surgical complications than those with morning surgery. This was attributed to the cyclical activity of Rev-Erbα, a protein expressed at high levels in the morning and that is diminished in the afternoon. By blocking the transcription of CDKN1a/p21, Rev-Erbα prevents apoptosis of cardiac cells, and thus protects patients [31]. Similarly, 80% of approved drugs target molecules in the body which have levels known to fluctuate according to predictable circadian rhythms. Statins as an example, inhibit 3-hydroxy-3-methylgluytaryl coenzyme A (HMB CoA) reductase to reduce cholesterol levels [32]. Peak enzyme activity is during the night and therefore it is recommended for patients to take their medication at night [33]. However, the importance that has been placed on the influence of the circadian rhythm on tissue homeostasis, sleep regulation, and behavior in the pharmaceutical industry has not been mirrored in the natural health and dietary supplement industries, leading to a gap in clinical trial design. Entraining the peripheral circadian clocks by time-restricted feeding has been found to improve health, underscoring the effect of circadian regulation on the efficacy of dietary supplements to achieve optimal health benefits. Given that most pharmacological preparations target molecules that exhibit circadian rhythms, the incorporation of a similar paradigm into clinical trials testing of THC and other cannabinoids must not be exempted from trial design. Clinical trial designs should be cognizant of how circadian misalignment at the molecular level may result in adverse health outcomes and could impede the efficacy of the intervention.

Exclusion criteria should reject participants with irregular sleep schedule and shift workers who have disrupted circadian rhythms and sleep [34]. Poor sleep hygiene among youth who are commonly perceived as a sleep-deprived population [35, 36] and the maturation changes that the adolescent brain undergoes around the age of 21 have a significant impact on circadian rhythm function [37, 38]. It is necessary to objectively monitor an individuals’ rest/wake cycle over a period of three weeks and reduce or eliminate the effect of first experimental night effects that generate non-representative results.

Clinical trials designed to test sleep quality, weight and mental health in individuals would require participants to maintain consistent bedtimes for at least 3 weeks prior to determine the level of plasma endocannabinoids [34]. Given that sleep quality influences plasma endocannabinoid levels of 2-AG [19], studies should capture the time of day when cannabis use occurs, have measures that can assess light exposure, track participant exercise through the use of actigraphic algorithms that can accurately detect distinct changes in activity, as well as include biologic measures of all potential drugs of abuse [34]. Few placebo-controlled clinical studies currently examine the plasma or serum level of endocannabinoids after administering healthy participants with THC, resulting in a lack of information regarding the acute effects of THC on endocannabinoids in humans. Including the measurement of the plasma levels of AEA and 2-AG along with the glucocorticoid and the noradrenergic system, particularly cortisol and norepinephrine levels, which in turn influence endocannabinoid levels, may be an essential component of clinical trial design.

The chronotype of the individual in a clinical study can predict addiction to cannabis products. Therefore, assessments using Morningness-Eveningness questionnaire of Horne and Osterberg, for example, can reveal whether the eveningness of the individual, associated with addiction and morningness, is associated with personality disorders [39], should be considered when designing a trial involving health and wellness products containing psychoactive substances.

Since the level of endocannabinoids is associated with food-seeking behavior and food intake, the method of cannabinoid intake during the study interval should reflect the practical nature of data collection, which will precisely define the method of drug administration and amount of cannabinoid ingested or inhaled [34]. However, health and wellness products are based on defined amounts of CBD or THC, and therefore clinical studies should be designed as such. Trial designs relying on self-reported time of day of ingestion should use reliable electronic and software applications designed for the purpose.

Limitations that can affect study design are the inclusion of participants varying widely in age to generalize the results. Cannabis administration should be compared to a placebo to experimentally delineate cannabinoid contribution to entrainment [34]. Such a design will identify confounding factors that affect circadian discrepancies among cannabis users and non-users [34].

In conclusion, evidence supports the influence of circadian rhythms on the levels of endocannabinoids, which in turn regulate the clinical efficacy of cannabinoids. While routes of administration have been discussed previously, optimization of the time of day of cannabis administration should be given equal, if not more, consideration when designing a study. Mitigating limitations associated with current clinical study designs by targeting a synchronized population for testing safety and efficacy of cannabis health and wellness products are essential for rational clinical study design in the cannabis space.
