# Erratum to “Changing the tone of clinical study design in the cannabis industry”

**DOI:** 10.1515/tnsci-2020-0110

**Published:** 2020-06-03

**Authors:** Joseph M. Antony, Alison C. McDonald, Farshid Noorbakhsh, Najla Guthrie, Mal Evans

**Affiliations:** KGK Science, London, Canada; Department of Immunology, School of Medicine, Tehran University of Medical Sciences, Tehran, Iran

In the published article “Changing the Tone of Clinical Study Design in the Cannabis Industry. Antony JM, McDonald AC, Noorbhaksh F, Guthrie N, Evans M. Transl Neurosci. 2020 Feb 13;11:4–9. doi: 10.1515/tnsci-2020-0002”, the spelling of one of the author’s name is given incorrectly. The Editorial Office of Translational Neuroscience would like to correct the author’s name. The editorial office apologizes for any inconvenience that it may have caused. JMA, ACM, FN, NG, and ME were the authors listed in the article. The correct author’s name should be Noorbakhsh F.

